# Psychology needs to get tired of winning

**DOI:** 10.1098/rsos.220099

**Published:** 2022-06-22

**Authors:** Gerald J. Haeffel

**Affiliations:** Department of Psychology, University of Notre Dame, Notre Dame, IN 46556, USA

**Keywords:** falsification, theory, predictions, philosophy of science, registered reports

## Abstract

Psychological science is on an extraordinary winning streak. A review of the published literature shows that nearly all study hypotheses are supported. This means that either all the theories are correct, or the literature is biased towards positive findings. Results from large-scale replication projects and the prevalence of questionable research practices indicate the latter. This is a problem because science progresses from being wrong. For decades, there have been calls for better theories and the adoption of a strong inference approach to science. However, there is little reason to believe that psychological science is ready to change. Although recent developments like the open science movement have improved transparency and replicability, they have not addressed psychological science's method-oriented (rather than problem-oriented) mindset. Psychological science still does not embrace the scientific method of developing theories, conducting critical tests of those theories, detecting contradictory results, and revising (or disposing of) the theories accordingly. In this article, I review why psychologists must embrace being wrong and how the Registered Report format might be one strategy for stopping psychology's winning streak.

## 1. Introduction

Nearly 100% of the published studies in psychology confirm the initial hypothesis [1]. This is an amazing accomplishment given the complexity of the human mind and human behaviour. Somehow, as psychological scientists, we always find the expected result; we always win!

Recently, however, the legitimacy of psychology's winning streak has been called into question. Major replication projects [2,3] show that only about half of psychological findings replicate. Further, there is evidence that psychology's winning streak may be due to cheating. Similarly to how baseball's homerun chase in the United States was fuelled by steroids (e.g. Bonds, McGuire, Sosa) and Lance Armstrong's Tour De France streak was aided by doping, psychology's winning streak may be the result of questionable research practices like p-hacking, HARKing, and piecemeal publication [4–6].

These problems sparked the open science movement [3] as well as a renewed interest in philosophy of science and the importance of theory-driven research. Classic articles about theory testing by scholars such as Meehl, Popper, and Platt are once again at the forefront of psychological discussions. This has led to an influx of new articles [7–10] arguing that psychology is plagued by poor theorizing. According to these accounts, psychological theories are too vague and unspecific, making them unfalsifiable. Thus, psychologists need new strategies for creating theories, such as computational modelling, construct validation, mathematical psychology, formal theory, and theory construction methodology. The take-home message of many of the articles is that psychologists do not have the tools or skill sets to create strong theories. They need better strategies to generate theories that have greater explanatory power for understanding human psychological processes.

Psychology would certainly benefit from better theories, and the proposed strategies may help. In fact, *any* strategy would likely help as work shows most published articles do not specify any theory. For example, a recent review found that only 15% of articles in *Psychological Science* (from 2009 to 2019) explicitly tested predictions derived from theories [11]. However, we contend the paucity of good theories is not because of a lack of strategies or training in how to create them. Theories are just educated guesses [12]. They do not need to be magnificent explanations of nature that emerge from advanced statistical techniques, formulized procedures, or even prior work. A good theory is just a highly specific, mechanistic guess that lends itself to being wrong and revised. Further, it is possible that the complexity of some of these proposed approaches may do more to discourage theory development than aid it [13].

This leads to the question—if creating a theory is as simple as making a risky guess, then why are there so few good ones in psychology? One reason is that psychology has stopped using the scientific method (i.e. (1) formulate theory/competing hypotheses, (2) devise a crucial experiment, (3) carry out the experiment, and (4) do it all again given the results). As noted by Platt [14, p. 348],

Science is now an everyday business. Equipment, calculations, lectures become ends in themselves. How many of us write down our alternative and crucial experiments every day, focusing on the exclusion of a hypothesis? We may write scientific papers so that it looks as if we had steps 1, 2 and 3 in mind all along. But in between, we do busy work. We become ‘method oriented’ rather than ‘problem oriented’.

In psychology, doing good science (winning) means publishing a lot, placing papers in prestigious journals, having a high h-index, and securing grant money. The goal is to publish as many papers as possible by discovering as many significant findings as possible. This approach to science has generated some important insights, but there is a difference between publishing results and publishing *meaningful* results. Meaningful results identify disagreements, lead to more specific theories and/or have useful applications. At some point, researchers have to ask, ‘What is this, what are we doing?' The problem is exemplified by the comments of the former head of NIMH: Thomas Insel: ‘I think I succeeded at getting lots of really cool papers published by cool scientists at fairly large costs—I think $20 billion—I do not think we moved the needle in reducing suicide, reducing hospitalizations, improving recovery for the tens of millions of people who have mental illness'.

Because psychology does not use the scientific method's iterative process to generate knowledge, the published findings do not build upon each other to create a deeper understanding of a problem. Instead, the literature comprises loosely connected findings in which ‘a part of the totality can be pulled out and attacked in isolation with seeming impunity' [15, p. 299]. Consequently, psychological science is operating in a largely unfalsifiable manner. The result is a lack of scientific progress.

Moreover, when a field is method-oriented rather than problem-oriented, there is no clear roadmap for improving scientific progress. In psychology, research agendas tend to be dictated by the latest fads and advances in statistics (e.g. multitrait multimethod modelling, data mining) and technology (e.g. fMRI, GWAS, TMS, EMA, VNS, etc.), which emerge in response to the ever-changing zeitgeists (e.g. behaviourism, cognitivism, decade of the brain, molecular genetics, big data). It is not that these advances are unimportant, but they should be a means to an end (i.e. tools for testing theories) rather than the primary focus of the science.

Contrast this with other areas of science (e.g. physics) where ‘winning' is characterized as progress towards understanding a problem. These areas use ‘strong inference' [14] in which scientists work to create specific, falsifiable theories and then expose their weaknesses (i.e. the scientific method). As noted by Platt [14], in these areas, journal articles regularly include sections in which the scientists specifically describe the conditions in which the theory would be invalid and then describe how the experiments were designed to rule out these alternative explanations. The idea is that over time, theories develop greater levels of verisimilitude as alternative explanations (and even entire classes of theories) are systematically eliminated. Theoretical refutations are met with excitement rather than ire and suspicion of scientific misconduct (although understandable at times in psychology given the prevalence of questionable research practices). For example, physicists recently found that they were incorrect in predicting the mass of a sub-atomic particle (W boson). This finding led scientists (Dr Mitesh Patel as quoted by the BBC News [16]) to make statements such as ‘the hope is that these cracks will turn into chasms and eventually we will see some spectacular signature that not only confirms that the Standard Model has broken down as a description of nature' and ‘we are all secretly hoping that this is really it, and that in our lifetime we might see the kind of transformation that we have read about in history books’.

Given the overarching mindset and value structure in psychology, there is little incentive for psychologists to partake in the ‘scientific game' [17] of creating strong theories and exposing their ideas to refutation. The field does not value this kind of research; rather, it endorses the idea that progress comes from publishing as many confirmations as possible; the more confirmations (i.e. wins), then the more accurate and valid the research area. Thus, why would anyone (other than for the love of the scientific process) create a highly specific theory and expose its weaknesses (or refute it), only to make the research unpublishable?

## 2. Why losing is better than winning (in science)

Sometimes when you win, you really lose, and sometimes when you lose, you really win

– *White* *Men Can't Jump* [18]

Recent articles [7–10] about theory building in psychology send an implicit message that science is about getting it right and that good theories provide ‘better' explanations and predictions. These articles gloss over the most fundamental aspect of science as discussed by Meehl [19,20], a self-proclaimed neo-Popperian—progress comes from disagreement. What differentiates science from non-science (i.e. the demarcation problem) is falsifiability [17]. If you can be wrong, then it is scientific; if you cannot be wrong, then it is not scientific. What makes a theory ‘good' is how easily it can be refuted; the more specific and fine grained a theory is, the more chance it can be wrong.

In science, refutations are more useful than confirmations. Even if psychology can address questionable research practices and the published findings are now ‘valid', it will still be impossible to conclude a theory is correct. This point is made by Popper's ‘all swans are white' example. In this example, validation of the white swan theory is pursued via confirmation; the greater the number of white swans detected, the better the theory. Despite all the confirmations, however, it is impossible to know if the theory is true as there may be yet to be discovered black swans (note, in a recent real-world example of this, researchers just documented the existence of a bright yellow king penguin [21] disconfirming the theory all king penguins are black and white). The point is that while it may be impossible to prove a theory, it is possible to be wrong. Empirical contradictions are powerful as they can refute a theory (or even a category of theories), which then leads to a revised formulation and new knowledge (from which the iterative learning process starts again). Learning only happens when we discover disagreements.

Psychology is finally finding black swans. A number of prominent theories have been called into question including ego depletion [22], the action-sentence compatibility effect [23], frontal asymmetry as a marker for depression [24], the growth mindset [25], and various priming effects [26,27]. In the absence of these refutations, progress in psychology was stagnant as researchers considered these (incorrect) theories as proven. Much like astrology, there was no new knowledge or progress despite the hundreds of positive results (i.e. white swans) published each month. The question now is whether psychologists will revise these theories to explain the contradictory results (i.e. propose theories that can account for the new information), or will they move on to more popular areas of psychology in which positive results are less scrutinized and easier to find.

## 3. The consequences of a scattered research literature

There are negative consequences to having a scientific literature comprising loosely connected research findings with no organizing framework and theoretical foundation. One consequence is having to rely on this literature to make predictions and policy decisions. Psychologists are quick to publish predictions about how humans will behave (e.g. vaccine hesitancy), the promise of genetic and biological breakthroughs, the widening of educational gaps, and factors influencing future mental illness. But, given the state of psychologic literature, how valid are these prophecies?

Recently, a prediction was put forth by some of the most prominent researchers in clinical psychology [28]. This ensemble of more than 30 researchers wrote a call-to-action article about the unparalleled effects COVID was going to have on rates of mental illness and the ‘widespread need for COVID-19-related mental health services for youth and adults'. They stated that it is ‘reasonable to assume that the pandemic will be associated with a substantial, sustained, and potentially severe “mental health curve” that, like the prevalence of the virus itself, will also need flattening given already insufficient mental health resources in the USA” (p. 414). They cited reasons such as an increase in the ‘already remarkably frequent use of digital media’, ‘unprecedented role confusion', and lockdown-induced loneliness, excessive substance use, and overexposure to marital discord. They also underscored the likelihood of increased risk of suicide. In response, they called for the ‘identification of psychologically active interventions that can be delivered at scale (cf. fluoride in the water) to build resilience and reduce risks among large segments of the population that may never find their way to a traditional psychotherapy session' (p. 421).

However, there is now growing evidence that there has not been a population-wide mental health crisis. It is possible that this situation changes, but two years after the start of COVID, a full-blown mental health crisis has not happened [29]. *The Lancet*'s COVID-19 Commission Mental Health Task Force [30,31], which reviewed data from almost 1000 studies conducted in nearly 100 countries, concluded: ‘A clear and consistent body of evidence suggests that psychological distress increased during the early months of the COVID-19 pandemic and that most (but not all) facets returned to pre-pandemic levels by mid-2020. While some components of subjective well-being showed signs of strain (e.g. increasing negative emotions), the data also reveal notable signs of resilience in life satisfaction, loneliness, social connection, and suicide'. There were several findings that contradicted the prophecies (table 1). Taken together, these findings suggest that clinical psychologists got it wrong (particularly compared with other predictions about COVID such as the widening educational divide [52] and vaccine hesitancy [53]). It is not that the pandemic has had no effect on mental health, but rather that clinical scientists were not able to predict what subgroups would be most affected (see Banks *et al*. [29] for further discussion). If clinical psychology is to take to ‘the airwaves (radio, cable news)' and ‘play a leadership role in guiding a national response for the foreseeable future' [28], then it needs to establish a track record of more accurate predictions. This is only possible if clinical scientists embrace being wrong and learn from it. As noted by Aknin [32], ‘the astonishing resilience that most people have exhibited in the face of the sudden changes brought on by the pandemic holds its own lessons. We learned that people can handle temporary changes to their lifestyle—such as working from home, giving up travel, or even going into isolation—better than some policy makers seemed to assume' (para. 12). It is not always easy to accept data that disagrees with our beliefs, but scientific progress depends on it.

**Table 1.** Mental health predictions and outcome data to date.

prediction	outcome
‘The current pandemic increases the risk for suicide in at least four ways that require a far greater investment in suicide science, along with new approaches to suicide screenings and imminent risk assessments across all types of clinical care'.	COVID did not lead to an increase in suicide [32–36]. According to Aknin *et al.* [32, para. 7], ‘real-time data from official government sources in 21 countries showed no detectable increase in instances of suicide from April to July 2020, relative to previous years; in fact, suicide rates actually declined slightly within some countries, including the USA'.
‘Social isolation and ongoing media coverage focusing on social-environmental threat may result in increased rumination and worry that drive biological processes such as inflammation. Social isolation and stay-at-home orders also may interfere with the ability to experience positive affect, apply social strategies for regulating affect, and use rewarding experiences to offset negative emotions'.	There were only modest changes in people's ratings of loneliness and social connectedness [37–40].
‘It is thus reasonable to assume that the pandemic will be associated with a substantial, sustained, and potentially severe “mental health curve” that, like the prevalence of the virus itself, will also need flattening'.	There is no evidence of a full-blown mental health crisis [29]. Ratings of life satisfaction did not decrease [32,41]; the average satisfaction rating for the COVID year was identical to the previous year.
‘Many adolescents have increased their already remarkably frequent use of digital media to compensate for the loss of in-person social interactions, yet emerging research suggests that digitally mediated social interactions may be distinct in form and psychological function from face-to-face experiences'.	Digital technology use during COVID-19 was not associated with depressive and anxious symptoms or suicidal ideation at the within- or between-person levels [42,43]. Also, there was a general downward trend over the course of the pandemic in pornography use [44].
‘Anxiety and depressive symptoms are likely to increase during the COVID-19 pandemic…the COVID-19 pandemic is also likely to precipitate substantial increases in depression'.	Despite disruptions in school and greater social isolation, adolescents did not report increases in suicidal thinking [45] and their levels of depression and anxiety were fairly stable [46]. There was even some evidence that teens have experienced decreases in depression and loneliness during the pandemic [47].
‘Older adults are uniquely vulnerable during COVID-19, both physically and psychosocially. This abrupt physical threat and loss of social resources may increase risk for loneliness, isolation, and depression among older adults'.	The elderly did not report the greatest increase in mental health symptoms [45]. A number of studies showed that negative mental health symptoms were less prevalent in older adults than younger adults [38,42,48].
Omission of prediction in this case. This finding was not anticipated.	Young females with children under 5 years of age reported the most mental health distress [49–51].

Indeed, failure to provide these kinds of checks and balances may lead to the kind of Sisyphean cycle found in digital technology as described by Orben [54]: ‘there is little impetus for the field to reflect about its own methodology and its place in the network of political, academic, and public spheres that drive this inefficient cycle. To ensure that psychology does not become an accomplice to a never-ending Sisyphean cycle of technology panics, the research area has to acknowledge the need for radical change. Psychologists need to recognize the increasingly prominent role they play in facilitating cycles of technology panics and consider whether what they are doing is bringing a net benefit to society and academia' (p. 1154).

In the case of the COVID call to action, it may even have detrimental effects for society. For example, there are potential problems with promoting a mental health crisis and recommending universal prevention interventions for ‘large segments of the population' [28, p. 421]. First, this ‘mass crisis’ messaging is not supported by existing data. Research shows that most people are resilient even when faced with high levels of stress [55–57]. Second, spreading the idea of a mental health crisis might pathologize normal psychological reactions to stress. Implicit in the ‘crisis’ message is that humans are supposed to be content and happy all the time. And being anxious or distressed, even during difficult times, means that one is unable to cope properly or indicates poor mental health. Further, the crisis messaging may lead people to hyper focus (and potentially misinterpret the severity of) their mental health symptoms. It might also exacerbate the negative forecasting errors people already make [58]. It is possible that if people are led to believe that increases in distress are indicative of mental illness, then they might flood the mental health system leading to fewer resources from those who really are in crisis. Finally, the recommendation for widespread mental health interventions (e.g. fluoride in the water) could do more harm than good. Research indicates that intervening too early or with people not at risk of mental health problems can disrupt the normal recovery processes that create resilience. For example, there is some evidence that interventions such as grief counselling and critical incident stress debriefing can be iatrogenic. As noted by Bonanno [59], ‘many individuals exposed to violent or life-threatening events will show a genuine resilience that should not be interfered with or undermined by clinical intervention' (p. 22). Supporting this claim, at least one study reported that nearly 40% of the individuals receiving grief treatments actually got worse relative to no treatment at all [60].

This is just one example of how a weak scientific knowledge base can make it difficult to generate accurate predictions. This same point was made by Ijzerman *et al*. [61] in response to recommendations made by a group of social psychologists [62] regarding the use social and behavioural science to improve the pandemic response. As noted by Ijzerman *et al*., ‘the way that social and behavioural research is often conducted makes it difficult to know whether our efforts will do more good than harm' (p. 1092). Indeed, if psychological science is to inform policy and help the public, then it likely needs to change its scientific approach to one of strong inference.

## 3. Registered reports: a new hope?

Researchers and philosophers have been discussing the problems with psychological science for over 50 years, and there is little reason to believe the field's approach to science will suddenly change for the better. But, one might ask, what about the open science movement? Indeed, this movement has led to the refutation of some theories (via non-replication) and the creation of new strategies for increasing scientific transparency (e.g. pre-registration, open data, article ‘badges’ for participating in open science practices). Although these improvements are necessary, they are not sufficient to fix psychology. They do not change the overarching mindset plaguing psychology, namely the pursuit of publishing results rather pursuing problems. Even if all the findings in psychology were replicable, it would not mean that the field was generating cumulative knowledge. Rather, we are simply adding more disparate results (although replicable in this case) to the literature. Indeed, as argued by Newell [15], ‘we never seem in the experimental literature to put the results of all the experiments together…my concern, to state it once more, is with how they will all add up' (p. 300).

Given that systemic change in psychology is unlikely, the best strategy may be to try to leverage the current value system to create incidental change. In other words, create a system that allows psychologists to maintain their ‘winning' mentality, but at the same time it makes for better science. We contend the best option for fixing psychology's ‘winning' problem is the Registered Report format in which articles are accepted or rejected prior to knowing the results of the study [63]. Scheel *et al*. [1] compared results reported in published Registered Reports with those of standard publications; they found 44% positive results in Registered Reports and 96% positive results in standard publications. This data is interesting because it helps disentangle competing hypotheses about why psychologists engage in questionable research practices. One explanation for the cheating is that psychologists is trying to protect their ideas; they simply do not want to be wrong. An alternative explanation is that psychologists love to get publications and need positive results to do so. The data from Registered Reports indicate that questionable research practices are about getting publications rather than saving ideas and theories. Psychologists are willing to be wrong as long as they can still get a publication. Thus, Registered Reports may be a powerful tool for fixing psychology; theories can lose while researchers continue to win.

It is possible that making Registered Reports the default publishing option will eventually move psychologists towards a more problem-focused (rather than method-oriented) mindset. Psychologists will no longer need to worry about obtaining positive results or oversell their findings because these factors will not determine if a manuscript is accepted for publication. Rather, manuscripts will be judged on the rationale for conducting the study and the ability of the design to test the hypotheses. This may ultimately force researchers to care more about the scientific process and the problems of interest. As noted by Platt [14], ‘the method-oriented man is shackled; the problem-oriented man is at least reaching freely toward what is more important’ (p. 351).

## 4. Conclusion

Recent articles [9,64,65] make a convincing case for the poor state of theory development (e.g. lack of precision and falsifiability) and the need for greater scientific transparency in psychology [3], but they fail to underscore why this is such a problem. Psychology needs to fix questionable research practices, but the motivation for doing so should not be to get *better* at winning. There seems to be the notion that if we create better theories and repair our methods (e.g. larger sample sizes, pre-registrations, open data, etc.), then we can continue to only publish positive results because we can be confident the findings are ‘accurate'. Although it is likely that greater transparency and methodological rigor will increase replication rates, it does not guarantee the theories are correct. Transparency and better theories may be necessary, but they are not sufficient for scientific progress. No amount of open science, new technology, and advanced statistical techniques and methods can fix psychology's winning mindset. These advances may lead to new insights and knowledge, but in the absence of strong inference, they do not sum to anything greater than their individual parts. The scientific landscape will continue to be a scattered, disjointed pile of research findings.

Psychology has learned far more from losing than winning. The field must stop the idea of progress by confirmation approach whereby if a study ‘works’, then we get to publish it, and if it does not work, then we hide it away. If psychology is to ever develop into a cumulative science, then it must get over the idea that being wrong is bad or a ‘crisis’ [66–68] and recommit to the scientific method.

## Data Availability

This article has no additional data.
